# An analysis of baseline data from the PROUD study: an open-label randomised trial of pre-exposure prophylaxis

**DOI:** 10.1186/s13063-016-1286-4

**Published:** 2016-03-24

**Authors:** David I. Dolling, Monica Desai, Alan McOwan, Richard Gilson, Amanda Clarke, Martin Fisher, Gabriel Schembri, Ann K. Sullivan, Nicola Mackie, Iain Reeves, Mags Portman, John Saunders, Julie Fox, Jake Bayley, Michael Brady, Christine Bowman, Charles J. Lacey, Stephen Taylor, David White, Simone Antonucci, Mitzy Gafos, Sheena McCormack, Owen N. Gill, David T. Dunn, Anthony Nardone, On behalf of the PROUD Study Group

**Affiliations:** MRC Clinical Trials Unit at UCL, Aviation House, 125 Kingsway, London, WC2B 6NH UK; HIV/STI Department, Public Health England, London, UK; Chelsea and Westminster Hospital NHS Foundation Trust, London, UK; The Mortimer Market Centre, Central and Northwest London NHS Foundation Trust, London, UK; Claude Nichol Centre, Royal Surrey Sussex County Hospital, Brighton, UK; Manchester Centre for Sexual Health, Central Manchester University Hospitals NHS Foundation Trust, Manchester, UK; St Mary’s Hospital, Imperial College NHS Foundation Trust, London, UK; Homerton University Hospital NHS Foundation Trust, London, UK; Ambrose King Centre, Barts Health NHS Trust, London, UK; Guy’s and St Thomas’ NHS Foundation Trust, London, UK; King’s College Hospital NHS Foundation Trust, London, UK; Sheffield Teaching Hospitals NHS Foundation Trust, Sheffield, UK; York Hospitals NHS Foundation Trust, York, UK; Heart of England NHS Foundation Trust, Birmingham, UK

**Keywords:** Pre-exposure prophylaxis (PrEP), Men who have sex with men (MSM), HIV prevention, Tenofovir, Truvada

## Abstract

**Background:**

Pre-exposure prophylaxis (PrEP) has proven biological efficacy to reduce the sexual acquisition of the human immunodeficiency virus (HIV). The PROUD study found that PrEP conferred higher protection than in placebo-controlled trials, reducing HIV incidence by 86 % in a population with seven-fold higher HIV incidence than expected. We present the baseline characteristics of the PROUD study population and place the findings in the context of national sexual health clinic data.

**Methods:**

The PROUD study was designed to explore the real-world effectiveness of PrEP (tenofovir-emtricitabine) by randomising HIV-negative gay and other men who have sex with men (GMSM) to receive open-label PrEP immediately or after a deferral period of 12 months. At enrolment, participants self-completed two baseline questionnaires collecting information on demographics, sexual behaviour and lifestyle in the last 30 and 90 days. These data were compared to data from HIV-negative GMSM attending sexual health clinics in 2013, collated by Public Health England using the genitourinary medicine clinic activity database (GUMCAD).

**Results:**

The median age of participants was 35 (IQR: 29–43). Typically participants were white (81 %), educated at a university level (61 %) and in full-time employment (72 %). Of all participants, 217 (40 %) were born outside the UK. A sexually transmitted infection (STI) was reported to have been diagnosed in the previous 12 months in 330/515 (64 %) and 473/544 (87 %) participants reported ever having being diagnosed with an STI. At enrolment, 47/280 (17 %) participants were diagnosed with an STI. Participants reported a median (IQR) of 10 (5–20) partners in the last 90 days, a median (IQR) of 2 (1–5) were condomless sex acts where the participant was receptive and 2 (1–6) were condomless where the participant was insertive. Post-exposure prophylaxis had been prescribed to 184 (34 %) participants in the past 12 months. The number of STI diagnoses was high compared to those reported in GUMCAD attendees.

**Conclusions:**

The PROUD study population are at substantially higher risk of acquiring HIV infection sexually than the overall population of GMSM attending sexual health clinics in England. These findings contribute to explaining the extraordinary HIV incidence rate during follow-up and demonstrate that, despite broad eligibility criteria, the population interested in PrEP was highly selective.

**Trial registration:**

Current Controlled TrialsISRCTN94465371. Date of registration: 28 February 2013.

**Electronic supplementary material:**

The online version of this article (doi:10.1186/s13063-016-1286-4) contains supplementary material, which is available to authorized users.

## Background

Pre-exposure prophylaxis (PrEP) has proven biological efficacy to reduce the sexual acquisition of human immunodeficiency virus (HIV). In 2012 the US Food and Drugs Administration approved the use of daily Truvada (tenofovir and emtricitabine) as PrEP [1] based on several placebo-controlled randomised trials [2–4].

In England, gay and other men who have sex with men (GMSM) are the population most likely to acquire HIV sexually, according to surveillance data routinely collected from sexual health clinics. Using the CD4 back calculation estimate of HIV incidence, 2470 men who have sex with men (MSM) were estimated to have acquired their HIV infection in the UK in 2013. This number has increased steadily from 2004 and now accounts for 51 % of all new HIV infections [5].

In addition to its biological benefits, the clinical delivery of PrEP currently requires contact with clinical services to enable regular testing for HIV and sexually transmitted infections (STIs), as well as access to behavioural interventions. Modelling suggests that introducing PrEP would have a large impact on the UK epidemic [6], in the context of provision to an appropriate population [7].

In England, sexual health for GMSM at risk of HIV is primarily provided by a network of over 200 open-access free clinics with 92,037 HIV-negative GMSM attending in 2013. To roll out PrEP in this setting, evidence of effectiveness that takes account of any change in risk behaviour is required [8]. This could only be captured in an open-label trial without a placebo control, ideally within a schedule and in a setting where PrEP could be delivered realistically (in terms of capacity and resources) [9]. Factors that could limit the population effectiveness of PrEP include changes in sexual behaviour [10] and the emergence of drug resistance [11]. In a nationally funded health service, it is also essential to consider the potential impact of funding diversion on other prevention activities and delivery of clinical services [12, 13].

The PROUD study demonstrated that the inclusion of PrEP gave a relative reduction of 86 % in the incidence of HIV, with no infections among participants taking PrEP, and refuted concerns that real-world effectiveness would be compromised [14]. HIV incidence in the study population was shown to be approximately seven times higher than the estimate for GMSM attending sexual health clinics in 2012. Here we present the baseline characteristics of the PROUD study population and place the findings in the context of national data returned from the sexual health clinics.

## Methods

The PROUD trial was designed to explore the real-world effectiveness of PrEP in which eligible HIV-negative GMSM received open-label tenofovir-emtricitabine (TDF-FTC) either immediately, in a risk reduction package, or after a deferral period of 12 months follow-up. The intention was of implementing this in sexual health clinics and with study procedures as close as possible to routine care in this setting. This paper reports the characteristics of participants recruited during a pilot phase, which aimed to establish the feasibility of a complete trial. However, the unexpectedly large number of HIV infections during the deferral period led to a recommendation from the Trial Steering Committee in October 2014 that all participants be offered PrEP. The findings on the effectiveness of PrEP were published and a larger trial is no longer required [14]. Trial procedures during the study are described in detail elsewhere [14].

The anticipated target for an adequately powered trial was 5000 (2500 per arm). This was based on an estimated incidence of 3/100 person-years during the first year in participants who were waiting to access Truvada and a 50 % reduction in incidence in those offered Truvada. An arbitrary 10 % target of 500 for the pilot phase was a pragmatic choice to guide as to whether 5000 participants could be enrolled over 2 years, according to the eligibility criteria in Table 1.

**Table 1:** Eligibility requirements

Inclusion criteria	Exclusion criteria
1. Born to male gender, age 18 years or older 2. Previously attended the enrolling clinic on at least one occasion 3. Completed a screen for HIV and STIs 4. HIV-negative by a routinely used assay within 4 weeks prior to or on the day of randomisation 5. Reported unprotected anal intercourse (UAI) on more than one occasion within the 90 days prior to randomisation 6. Likely, in the opinion of the volunteer, to have UAI in the next 90 days 7. Willing and able to comply with the visit schedule throughout the follow-up period 8. Willing and able to provide written informed consent	1. An acute viral illness that could be due to HIV seroconversion 2. Any contraindications to Truvada according to the current package insert 3. Treatment for hepatitis B infection indicated or ongoing 4. Unlikely, in the opinion of the clinician, to comply with the randomised allocation

*HIV* human immunodeficiency virus, *STI* sexually transmitted infection

Potentially eligible GMSM were identified during routine attendances at 13 sexual health clinics in England, 8 in London and 5 outside (Birmingham, Brighton, Manchester, Sheffield and York). Participants with regular sexual partners (in the opinion of the potential volunteer) who also met eligibility requirements were encouraged to enrol at the same time and both partners were randomly allocated to the same trial arm, minimising the possibility of PrEP being shared. Posters and electronic screens in participating sexual health clinics, as well as advertisements on social media, helped to promote the study. Business cards and leaflets advertising the study were also handed out by community organisations during outreach activities, including efforts to raise awareness of PrEP amongst GMSM. There was no financial payment for participants joining the study, nor were travel costs or other expenses paid for.

A screening visit was not required as the eligibility data on HIV are collected routinely. The Participant Information Sheet was shared with volunteers prior to enrolment. The research team at the clinic determined eligibility through a structured discussion with the volunteer and written informed consent was collected prior to enrolment. Eligible participants were randomised using a web-based tool incorporated within the database at each clinic. At the enrolment visit participants were asked to self-complete, in private, two paper baseline questionnaires (Additional files 1 and 2) on separate booklets collecting information on: demographics, sexual behaviour and lifestyle in the last 30 and 90 days; perception of HIV risk at the last condomless sex act; risk management strategies; past history of STIs; drug and alcohol use; depression severity captured by the Patient Health Questionnaire-9 (PHQ-9) [15]; motivation for taking part in the study; and perceptions regarding adherence to taking a daily pill. The questionnaires were derived from other studies in MSM populations and were being tested as part of the pilot phase. Questionnaires took less than 30 minutes to complete and were placed in a sealable envelope and sent to the Medical Research Council Clinical Trials Unit (MRC CTU) at University College London (UCL) for data entry without clinic staff seeing the responses.

A screen for STIs was performed, if indicated, according to routine clinical practice. A sample for antibody/antigen HIV testing was collected on the day of enrolment (Table 1).

In NHS sexual health clinics, basic demographic, diagnostic and service data are also returned for each attendance and collated by Public Health England, using the genitourinary medicine clinic activity database version 2 (GUMCADv2). GUMCADv2 is a pseudo-anonymised patient-level electronic dataset collecting information on diagnoses made and services provided by genitourinary medicine (GUM) clinics (level 3) and other commissioned level 2 (non-GUM) sexual health services [16]. For this analysis, data were extracted from GUMCADv2 on GMSM who were HIV-negative or of unknown status from all clinics in England between January 2013 and December 2013. Data were extracted on key demographics including age, ethnicity and place of birth, STI diagnoses by number of GMSM, as opposed to by GMSM attendances, (pharyngeal, urethral and rectal chlamydia and gonorrhoea, primary secondary and early latent syphilis, hepatitis B and C infections, lymphogranuloma venereum (LGV)), HIV tests (number of HIV tests and average number per GMSM), and episodes of post-exposure HIV prophylaxis (PEP) among GMSM.

The data were analysed using Stata 13.1 [17]. Comparisons of categorical data were conducted using a χ^2^ test or a two-tailed Fisher exact test where numbers were less than five in any group. Participants recruited within London were compared to those recruited outside of London to determine differences in baseline demographics, sexual behaviour and lifestyle. Continuous variables were compared using a Mann-Whitney *U* test. Multiple component analysis (MCA) was used to determine whether responses to questions where participants could select multiple answers decomposed into distinct groups [18].

The PROUD study protocol was approved by London Bridge Research Ethics Committee, the Medicines and Healthcare products Regulatory Agency and each participating hospital trust (Chelsea and Westminster Healthcare NHS Foundation Trust, London, UK; Brighton and Sussex University Hospitals NHS Trust, Brighton, UK; Homerton University Hospital NHS Foundation Trust, London, UK; Central Manchester University Hospitals NHS Foundation Trust, Manchester, UK; Imperial College Healthcare NHS Foundation Trust, London, UK; Sheffield Teaching Hospitals NHS Foundation Trust, Sheffield, UK; York Teaching Hospital and Hull York Medical School, York, UK; Barts Health NHS Trust, London, UK; King’s College Hospital NHS Foundation Trust, London, UK; Guy’s and St Thomas’ NHS Foundation Trust, London, UK; and Heart of England NHS Foundation Trust, Birmingham, UK).

## Results

### Recruitment

**Fig. 1:**
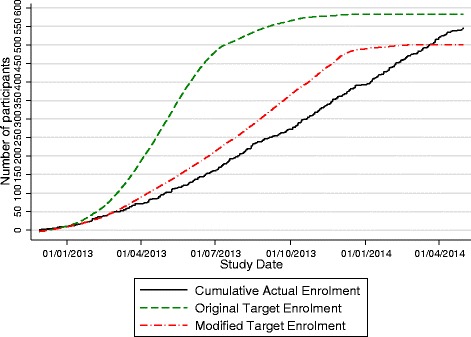
Cumulative recruitment by time

**Fig. 2:**
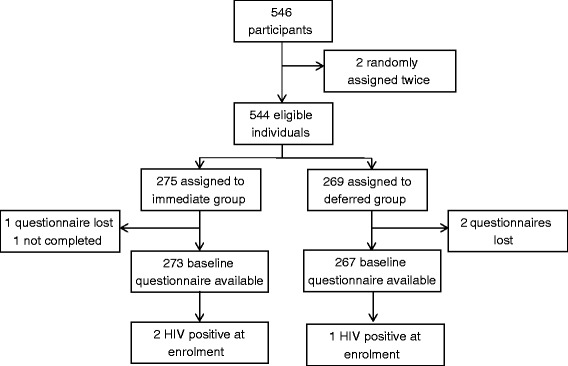
Flow diagram of enrolment

The PROUD pilot study enrolled the first participant on 27 November 2012. The pilot study was initially expected to fully recruit within 6 months, but this target was modified in April 2013 due to slow recruitment, attributed to lack of study resources delaying clinic activation, and low awareness of PrEP in the community [19]. The modified target was that full recruitment would be reached in April 2014. The two cumulative targets and actual recruitment are shown in Fig. 1. Recruitment was stopped at the end of April 2014 after 544 participants were enrolled. Two participants enrolled twice to access PrEP and were analysed in their original trial arm, the deferred group (Fig. 2). In total, 19 participants enrolled with their partner in the trial and were randomised to the same trial arm.

### Baseline demographics

Data from the self-completed questionnaire were available for 540/544 participants (99 %); missing questionnaires were either lost (*n* = 3) or not completed by participants (*n* = 1).

Baseline demographic data are shown in Table 2. The median age of participants was 35 (interquartile range (IQR): 29–43) with an age range of 18–75, and the majority of participants were of white ethnicity (81 %). Participants who described themselves as of an ‘Other’ ethnicity defined themselves as Chinese (*n* = 11), Latin American (*n* = 10), Arabic (*n* = 5), Asian (*n* = 5) or mixed race or other (*n* = 5).

**Table 2:** Baseline demographic data

	Number	%
Age		
18–25	54	10
25–35	210	39
35–45	178	33
>45	98	18
Ethnicity		
White/Irish	439	81
Indian/Pakistani/Bangladeshi	18	4
Black Caribbean/Black-African/Other	21	4
Mixed ethnic group	24	4
Other	36	7
Missing	2	0
Born in UK		
No^a^	217	40
Yes	322	60
Missing	1	0
Sexuality		
Gay/homosexual	514	95
Bisexual	16	3
Straight/heterosexual	6	1
Missing	4	1
Maximum education		
No qualifications	14	3
O-levels/GCSEs/Equivalent	60	11
A-levels/Equivalent	87	16
University degree or above	327	61
Still in full-time education	19	4
Vocational training/Other qualifications	32	6
Missing	1	0
Circumcised		
No	380	70
Yes	156	29
Missing	4	1
PHQ-9 depression severity		
Minimal	381	71
Mild	88	16
Moderate	27	5
Moderately severe	16	3
Severe	6	1
Missing	22	4
Current relationship status		
Yes, I live with my partner	160	30
Yes, I am in a relationship but do not live with my partner	86	16
No, I’m not in an ongoing relationship	293	54
Missing	1	0

^a^37% Europe, 13 % Africa, 12 % Central/South America, 12 % Asia, 12 % North America, 10 % Australasia and 4 % missing

Typically participants were highly educated, 61 % were educated at a university degree level or above. Participants were predominantly employed either full-time (72 %) or part-time (10 %). Of all participants, 217 (40 %) were born outside the UK. In total, 156 (29 %) participants were circumcised and circumcision was significantly higher in participants born outside the UK (*n* = 84; 39 % versus *n* = 72; 23 %; *p* <0.001). The majority of participants described their sexuality as gay/homosexual (*n* = 514; 95 %), 16 (3 %) as bisexual and 6 (1 %) as straight/heterosexual. Only one participant defined themselves as transgender. A prevalence rate for moderate to high severity of depressive symptoms of 9.5 % was identified, lower than those observed in HIV-1-positive MSM in the Antiretrovirals, Sexual Transmission Risk and Attitudes (ASTRA) study (27.0 %) [20]. The prevalence of a major depressive disorder was 5.0 %, higher than the 2.5 % observed in a general European male population [21]. Almost half (46 %) of the participants described themselves as being in an ongoing relationship and 160 (30 %) were currently living with their partner.

### Sexually transmitted infections

STI data from the clinic interview (self-reported lifetime diagnoses), self-completed questionnaire (self-reported diagnoses in the last 12 months) and baseline STI test are shown in Table 3. Self-reported data on STI history were available for 515/544 (94 %) participants but the exact denominators differed by STI. A diagnosis of any STI in a participant’s lifetime was reported in 473/544 (87 %) participants. This was frequently urethral gonorrhoea (*n* = 216; 40 %), oral gonorrhoea (*n* = 175; 32 %), rectal gonorrhoea (*n* = 174; 33 %), urethral chlamydia (*n* = 173; 32 %) and genital warts (*n* = 168; 31 %). Participants reported being screened for STIs a median (IQR) of 3 (2–4) times in the previous 12 months. One or more STI diagnoses in the previous 12 months was reported in 330/515 (64 %) participants, in particular: rectal gonorrhoea (*n* = 126; 26 %), oral gonorrhoea (*n* = 121; 25 %), urethral gonorrhoea (*n* = 112; 24 %) and rectal chlamydia (*n* = 99; 21 %). At enrolment 47/280 (17 %) participants screened, based on clinical indication, were diagnosed with an STI. These were mostly oral gonorrhoea (*n* = 13), syphilis (*n* = 13), rectal gonorrhoea (*n* = 12) and rectal chlamydia (*n* = 10) diagnoses. In addition, three participants were found to be HIV-1-positive despite having an HIV-negative test in the 4 weeks prior to randomisation. These participants are included in these analyses of baseline data.

**Table 3:** Self-reported history of, and diagnosed sexually transmitted infections at enrolment

	Self-reported diagnoses (lifetime)^a^		Self-reported diagnoses (last 12 months)^b^		Diagnosed at enrolment	
	*N*	%	*N*	%	*N*	%
Rectal gonorrhoea	174/532	33	126/478	26	12/251	5
Urethral gonorrhoea	216/538	40	112/480	24	2/256	1
Oral gonorrhoea	175/539	32	121/483	25	13/255	5
Rectal chlamydia	160/533	30	99/470	21	10/248	4
Urethral chlamydia	173/538	32	80/478	17	3/255	1
Oral chlamydia	63/523	12	60/471	13	3/244	1
Lymphogranuloma venereum (LGV)	15/127	12	10/458	2	0/7	0
Syphilis	110/537	20	49/473	10	13/237	5
Hepatitis C	9/451	2	3/464	1	0/132	0
Genital warts	168	-	45/472	10	6	-
Genital herpes	73	-	25/464	5	10	-

Notes

^a^the data for lifetime diagnoses were collected by staff during the enrolment interview and accounted for conditions that were never tested for

^b^the data for diagnoses in the last 12 months were reported by participants on the questionnaire who were invited to indicate ‘yes’ or ‘no’ for each infection. In the event that no answer was returned, they were not included in the denominator for that infection

### Reported sexual risk management strategies and risk perception

Participants had attended a clinic a median (IQR) of 3 (2–4) times in the past 12 months for an HIV test. A course of PEP had been prescribed to 184 (34 %) participants in the past 12 months with 71 (13 %) having more than one course.

Overall 50 % of participants reported more than one strategy for managing their risk of contracting HIV. The following strategies were reported: using condoms for anal sex (*n* = 209; 39 %); choosing partners based on their negative HIV status (*n* = 199; 37 %); strategic positioning (being ‘on top’ if they are unsure of partner’s HIV status) (*n* = 157; 29 %); asking partners to use a condom for anal sex (*n* = 134; 25 %); seeking partners who are known to be on HIV treatment (*n* = 134; 25 %). Only 75 (14 %) participants reported that they did not think about risk reduction strategies. MCA highlighted two clear groups: 113 participants who exclusively reported condom use (self or partner) and 206 who reported serosorting or strategic positioning strategies. The other 131 participants used a combination of these strategies.

Participants were asked to self-categorise, into one of five groups, their perceived risk of contracting HIV if they have anal sex without using a condom: 8 (2 %) described themselves as at no risk; 146 (27 %) at little risk; 258 (49 %) as somewhat at risk; 85 (16 %) as at high risk; and 34 (6 %) at very high risk. Participants who considered themselves somewhat at risk or higher (*n* = 377) were more likely to have been diagnosed with syphilis, hepatitis C, rectal gonorrhoea or rectal chlamydia in the past 12 months (*n* = 151; 40 %) than those who considered themselves at little risk or lower (*n* = 46; 28 %; *p* = 0.009).

### Sexual risk behaviour

In the last 90 days, participants reported a median (IQR) of 10 (5–20) partners with a range of 1 to >100. Of these, a median (IQR) of 2 (1–5) were condomless partners where the participant was receptive (bottom), and a median (IQR) of 2 (1–6) were condomless partners where the participant was insertive (top). A median (IQR) of 7 (2–15) partners were new (partners a participant had not had sex with before). In the last 90 days, 55 (10 %) participants had 40 or more partners and 59 (11 %) participants had more than 10 condomless partners where the participant was receptive and 77 (14 %) had more than 10 condomless partners where the participant was insertive.

Participants reported a variety of reasons for not using a condom at their last condomless anal sex act: it is more enjoyable without a condom (*n* = 349; 65 %); I don’t like using condoms (*n* = 268; 50 %); he doesn’t like using condoms (*n* = 178; 33 %); condoms weren’t discussed (*n* = 140; 26 %); I was under the influence of drugs (*n* = 128; 24 %) or alcohol (*n* = 113; 21 %); I didn’t consider myself at risk of HIV (*n* = 115; 21 %); and we don’t use condoms with each other but do with other partners (*n* = 86; 16 %). MCA did not decompose responses to reasons for non-condom use into clear groups. At the last condomless anal sex act 239 (45 %) participants thought their partner was HIV-negative; 146 (28 %) thought their partner was HIV-positive and on treatment; 118 (22 %) did not know the HIV status of their partner; and 28 (5 %) thought he was HIV-positive and either not on treatment or did not know.

Among the 467 participants who reported sexual behaviour in both the last 30 days and the last 90 days at enrolment, participants typically reported more partners per 30 days in the last 30 days (median = 5; IQR = 2–10) compared to the last 90 days (median = 3; IQR = 2–7; *p* <0.001). This pattern was also consistent for the number of condomless partners where the participant was receptive (median (IQR) = 1 (0–3) versus 1 (0–1); *p* <0.001) and insertive (median (IQR) = 1 (1–3) versus 1 (0–2); *p* <0.001).

Participants in the PROUD pilot study reported their recreational drug use in the past 3 months: 394 (73 %) had used recreational drugs during this period. Poppers and Viagra were frequently used (*n* = 262; 49 % and *n* = 223; 41 % respectively). Other drugs used included: mephedrone (*n* = 197; 36 %), GHB/GBL (gamma-hydroxybutrate or gamma-butyrolactone) (*n* = 169; 31 %), cocaine (*n* = 139; 26 %), cannabis (*n* = 128; 24 %), crystal methamphetamine (*n* = 98; 18 %), ecstasy (*n* = 90; 17 %), ketamine (*n* = 89; 16 %) or some other drug (*n* = 92; 17 %). Drugs commonly associated with drug use in a sexual context (ChemSex: mephedrone, GHB/GBL or crystal methamphetamine) were used by 231/525 (44 %) participants in the past 3 months.

### London centres compared to out-of-London centres

Participants enrolled in the eight London-based clinics (*n* = 375; 69 %) were compared to participants enrolled in the five out-of-London clinics (*n* = 165; 31 %) to evaluate differences in baseline demographics, STIs and sexual risk behaviour. Participants in London were less likely to be white (75 versus 86 %; *p* = 0.003) and more likely to be Black-African, Black-Caribbean or mixed race (10 versus 4 %; *p* = 0.023). Participants in London were similar in age (median = 36 versus 35 years; *p* = 0.37) to out-of-London participants. London participants were more likely to be born outside the UK (50 versus 18 %; *p* <0.001) and more likely to be university educated (69 % versus 42 %; *p* <0.001). London participants had more HIV and STI tests in the last 12 months (mean of 3.5 versus 2.9; *p* <0.001 and mean of 3.1 versus 2.7; *p* = 0.006 respectively) and more courses of PEP in the past 12 months (mean of 0.64 versus 0.41; *p* = 0.022). London participants reported more diagnoses of STIs in the last 12 months than out-of-London participants (*n* = 243, 67 % versus *n* = 88, 56 %; *p* = 0.018) and had significantly more lifetime STI diagnoses (*n* = 342, 91 % versus *n* = 131; 80 %; *p* = 0.001). Finally, London participants also reported a higher total number of partners in the last 90 days (mean = 17.3 versus 13.3; median = 10 versus 10; IQR = 5–20 versus 4–18; *p* = 0.023) and significantly more condomless partners where the participant was insertive (mean of 5.1 versus 3.7; median = 2 versus 1; IQR = 1–6 versus 1–5; *p* = 0.029). However, there was no significant difference between London and out-of-London participants in the number of condomless partners where the participant was receptive (mean of 4.1 versus 3.7; median = 2 versus 2; IQR = 1–4 versus 1–4; *p* = 0.54) or the number of new partners (mean of 11.9 versus 9.3; median = 7 versus 6; IQR = 2–15 versus 1–11; *p* = 0.15). London participants were more likely to have used recreational drugs in the past 3 months (*n* = 287, 79 % versus *n* = 107, 66 %; *p* = 0.003).

### GUMCAD clinic comparison

PROUD participant data were compared to data from 92,307 HIV-negative GMSM who attended a GUMCAD clinic in 2013, of which 40,493 HIV-negative GMSM were seen at participating PROUD clinics [22]. There were significant differences in age between PROUD participants and GUMCAD attendees, with more participants in PROUD aged 34–44 (33 % versus 22 %; *p* <0.001) and fewer aged 20–24 (9 % versus 19 %; *p* <0.001). The ethnicity of PROUD participants appeared similar to GUMCAD attendees (81 % versus 80 % white ethnicity). PROUD participants were more likely to be born outside the UK compared to GUMCAD attendees (40 % versus 28 %; *p* <0.001), but were similar to GUMCAD attendees at PROUD clinics (40 % versus 43 %; *p* = 0.22). PROUD participants were more likely to have been recruited from London than the general GUMCAD clinic attendees (70 % versus 51 %; *p* <0.001). PROUD study participants had attended clinic a mean of 3.27 times in the past 12 months for an HIV test, compared to a mean of 1.13 for HIV-negative MSM attending GUMCAD clinics in 2013.

Whilst not directly comparable measurements, the number of STI diagnoses reported by PROUD participants is high in comparison to GUMCAD attendees. Rectal chlamydia was diagnosed in 2454 (3 %) GUMCAD attendees in 2013 and 2244 (2 %) were diagnosed with rectal gonorrhoea. There were a total of 585 primary syphilis diagnoses, 87 hepatitis C diagnoses and 107 LGV diagnoses in HIV-negative GMSM attending GUMCAD clinics in 2013, all less than 1 %. PEP use also appears high among PROUD participants compared to the general GMSM population in England: in GUMCAD clinics in 2013, there was a total of 4133 PEP prescriptions in 92,037 HIV-negative GMSM (4.5 %).

## Discussion

The PROUD study is the first randomised entirely open-label study of PrEP with a deferral design that aims to explore its real-world effectiveness, taking account of changes in behaviour that may follow the use of a drug known to reduce the risk of contracting HIV.

The baseline data presented here indicate that the GMSM who enrolled in the PROUD study are at substantially higher risk of acquiring HIV infection sexually than the overall population of GMSM attending sexual health clinics in England. Our data demonstrate that the PROUD cohort had several key indicators to support the observation that they were at high risk of HIV acquisition, particularly those recruited through the London centres. This is consistent with data suggesting that people living in London tend to have poorer sexual health outcomes [23, 24], are a population with higher rates of partner change and higher use of ChemSex drugs [25]. These indicators could inform discussions with potential PrEP users in the UK. Further analysis is required to establish whether these indicators are predictive of HIV infection among the participants who seroconverted during the study.

There is a striking excess of reported STIs in the 12 months preceding baseline in the PROUD cohort compared to the number of infections diagnosed in the general HIV-negative GMSM population attending clinics in 2013. This difference is not directly comparable, due to participants’ 12-month history of STIs not all occurring in 2013, and because diagnosed STIs are per clinic whilst reported STIs may span several clinics that a participant visits. Nonetheless, this difference suggests that PROUD participants have particularly high rates of STIs. Of note, a high proportion of the PROUD cohort reported previous rectal infections. Rectal infections, particularly with gonorrhoea and chlamydia, have been associated with higher risk of HIV infection and it has been suggested that a diagnosis of rectal STIs may be used as one of a number of criteria for actively recommending PrEP to GMSM clinic attendees [26]. Despite this excess of STIs at baseline, only 22 % of participants considered themselves to be at high or very high risk of HIV in general when they last had anal sex without using a condom. This highlights a limitation of this question as perceived HIV risk at the last anal sex act without a condom will vary by partner and may differ from a participant’s overall perceived risk of acquiring HIV.

Participants also reported high numbers of sexual partners, particularly condomless sex partners. The PROUD cohort reported a higher median number of partners in the preceding 90 days, compared to that reported elsewhere. For example, PROUD participants reported 10 partners versus 5 in the European EMIS survey of MSM [27] and versus 3 in the US National Behavioural Surveillance system survey [28]. In the Pre-exposure Prophylaxis Initiative (iPrEx) study, eligible GMSM reported an average of 7 sexual partners in the past 3 months at baseline and an average of 18 sexual partners in the past 12 months [2]. The median number of condomless sex partners reported in PROUD appears high in relation to European and US surveys. In EMIS, 58 % of GMSM reported at least one instance of condomless anal sex in the past 12 months [27]. In a US survey, white and black GMSM reported a median of two condomless anal sex partners in the past 12 months [29]. Further work should compare the distribution of the number of partners since the median is of limited value.

There is concern about the role of recreational drugs in facilitating condomless anal sex, leading to an increased number of partners and the possibility of rectal trauma after protracted periods of sexual activity. A survey of ChemSex (sex under the influence of drugs) in south London found that a third of men reported difficulty in negotiating safe sex whilst under the influence of drugs [25]. Although PROUD participants were not asked specifically about ChemSex, their use of recreational drugs in the past 3 months, particularly drugs associated with ChemSex such as mephedrone, crystal methamphetamine and GHB/GBL, was much higher than previously reported in national Internet surveys of GMSM [30] and the general population in England and Wales [31].

The study population reported an eight-fold higher use of PEP for sexual exposure in the past year when compared to the national surveillance data, with 13 % using it more than once. The costs associated with PEP are substantial and include drug costs and frequent clinic visits. Cost-effectiveness analyses need to be conducted to determine if a PrEP programme could reduce costs compared to PEP in a population with this level of PEP usage.

PROUD participants used several other risk reduction strategies such as condoms, serosorting and seropositioning. However, despite active risk management, including using strategic positioning, three participants had a reactive HIV antigen-antibody test at baseline, despite a non-reactive antibody point-of-care test. These recently acquired infections underscore the failure of existing risk reduction strategies to prevent infection. This is not surprising as the range of risk reduction strategies employed prior to enrolment were selective, either selective condom use or selection of partner and type of sex to have without a condom. Follow-up data from PROUD will provide an opportunity to assess how men fit PrEP into their existing strategies.

Previous cost-effectiveness studies for PrEP suggested that with current drug costs a daily PrEP programme will only be of cost-benefit if provided to the population most at risk of HIV [32–35]. Data from PROUD will inform decisions about commissioning of PrEP in England [36] and UK cost-effectiveness analyses [37, 38]; these concluded that daily PrEP, in a medium-sized PrEP rollout of 5000 GMSM, would only become cost-effective if current drug prices are substantially reduced. The baseline data suggest that it is feasible to deliver an effective PrEP programme through the sexual health clinic network. PROUD was a pilot study with very limited resources and recruitment depended not only on clinical providers identifying GMSM who would benefit from PrEP, but also GMSM recognising their need for additional options and informing others in their social and sexual networks.

There are some limitations to this analysis. Although data were available for the majority of participants at baseline, a small proportion of baseline data were missing. Efforts were made to contact all of these participants to collect basic demographic data. However, we did not retrospectively collect behavioural data as this would have been subject to recall bias. Recall bias may also explain the difference in 90-day versus 30-day risk assessment findings, with the suggestion that 90-day risk assessment may underestimate more recent risk.

The PROUD population were highly educated; they were older and more likely to have been born outside the UK compared to the general GMSM population attending GUM clinics in England, although were as equally likely to have been born outside the UK as attendees from the sexual health clinics in which they were recruited. The PROUD population has a similar median age to the age of first diagnosis among GMSM in England, which was 34 years in 2012 [39]. Due to the time lag between infection and diagnosis, this suggests that GMSM accessing PrEP in PROUD are also older than the age of the general GMSM population who seroconvert.

Black, Asian and minority ethnic (BAME) GMSM populations were similarly represented in the PROUD cohort compared to the population attending the same GUM clinics in England but had higher representation than the general GMSM population in England. Data from the US, UK and Canada suggest that this ethnic group is at highest risk of acquiring HIV and would benefit from PrEP. This needs to be explored further in the UK context where there are fewer disparities in access to health care [40] but where differences in the use of services remain [41].

PrEP was only offered in the PROUD study in major urban centres, and may not have been accessible to those living in more rural areas and in some parts of the country where there were no PROUD centres. In addition, the process of informed consent, randomisation to wait a year, and participation in a study, in general, could have deterred many eligible GMSM from enrolling. This could have contributed to the initial delay in recruitment to the study and forthcoming quantitative and qualitative work will describe the acceptability of the study in more detail. There were also unanticipated delays getting all 13 recruitment centres operational. Nonetheless, the rate of recruitment increased over time as information about the trial spread via word of mouth and the eventual population recruited were at high risk of HIV, so benefited greatly from the offer of PrEP.

Participants who enrolled in the PROUD study knew that PrEP had proven biological efficacy and these results demonstrate that the population interested in PrEP are likely to be highly self-selective for those at high risk of HIV acquisition. We expect similar effectiveness in populations with a lower risk of sexually acquiring HIV if there are similar levels of adherence, although our findings suggest that populations at low risk of HIV or with low rates of STIs may be less interested in the offer of PrEP if it becomes accessible as part of the NHS. Nonetheless, there were some participants in PROUD who had never reported an STI during their lifetime and further research is ongoing examining any changes in sexual risk behavior and STI diagnosis in this group.

## Conclusion

Baseline data from the PROUD pilot study suggest that it attracted a population of GMSM at high risk of HIV, reflected in high rates of STIs and higher risk sexual behaviours. These findings contribute to explaining the extraordinary HIV incidence rate during follow-up. Despite broad eligibility criteria, the population interested in PrEP were highly selective and substantially benefited from access to PrEP.

## Additional files

Additional file 1: Baseline Sexual Behaviour Questionnaire. (DOC 155 kb) Additional file 2: Adherence and Sexual Behaviour Questionnaire. (DOC 95 kb)

## Abbreviations

ASTRA: Antiretrovirals, Sexual Transmission Risk and Attitudes
BAME: Black, Asian and minority ethnic
ChemSex: the use of crystal methamphetamine, GHB/GBL or mephedrone either immediately before, or during sex
FTC: emtricitabine
GBL: gamma-butyrolactone
GHB: gamma-hydroxybutrate
GMSM: gay and other men who have sex with men
GUM: genitourinary medicine
GUMCAD: genitourinary medicine clinic activity database
HIV: human immunodeficiency virus
iPrEx: Pre-exposure Prophylaxis Initiative
IQR: interquartile range
LGV: lymphogranuloma venereum
MCA: multiple correspondence analysis
PEP: post-exposure prophylaxis
PrEP: pre-exposure prophylaxis
STI: sexually transmitted infection
TDF: tenofovir
UK: United Kingdom
US: United States
