# Supplementary material for: An analysis of baseline data from the PROUD study: an open-label randomised trial of pre-exposure prophylaxis
Source: Trials. 2016 Mar 24;17:163. doi: 10.1186/s13063-016-1286-4 (PMC4806447; doi:10.1186/s13063-016-1286-4)
Supplement: Additional file 1: — Baseline Sexual Behaviour Questionnaire. (DOC 155 kb) [file 13063_2016_1286_MOESM1_ESM.doc]

**PROUD**

**Baseline Sexual Behaviour Questionnaire**

| Trial no: | Initials: | Date of birth: | Date form completed: |
| --- | --- | --- | --- |

**Patient Questions**

1. **Are you:** Male  Transgender
2. **How would you describe your sexuality?**

   Gay/Homosexual  Straight/heterosexual  Bisexual  Other (please specify) ____________
3. **To which of these ethnic groups do you consider yourself to belong?**

| White | Chinese | Irish | Irish Traveller |
| --- | --- | --- | --- |
| Indian | Pakistani | Bangladeshi | Black Caribbean |
| Black African | Black Other | Mixed Ethnic Group_______ | Other ___________ |

1. **Were you born in the UK?** Yes  No  *(If yes skip to question 6)*
2. **If no, which country were you born in? ____________**
3. **What is your date of birth? __/__/____** *(please fill as DD/MM/YYYY)*
4. **At what level did you complete your education?**

Finished education with no qualifications

O levels/GCSEs (or equivalent qualifications at age 16)

A levels (or equivalent qualification at age 18)

University degree or above

Still in full-time education

Vocational Training

Other qualifications *(please specify)* __________

1. **What is your current work situation?**

Employed or self-employed FULL-TIME (at least 30 hours a week)

Employed or self-employed PART-TIME (less than 30 hours a week)

Part/full time student/education/training

Unemployed

Retired

Other *(please specify)*__________

1. **Are you currently in an ongoing relationship with a partner (wife/husband or civil partner or girlfriend/boyfriend)?**

Yes, I am in a relationship and living with my partner

Yes, I am in a relationship but not living with my partner

No, I am not currently in an ongoing relationship with a partner

1. **Have you been circumcised?** Yes  No

**Sexual Behaviour Questions**

1. **With how many different men have you had either been bottom (receptive, passive, he fucked you) or top (insertive, active, you fucked him) during anal sex in the last 90 days?**
2. **With how many different men have you been bottom (passive) during anal sex in the last 90 days?**  *(If 0 please skip to question 16)*
3. **Of the men in question 12, with how many were you bottom without using a condom?**  *(If 0 please skip to question 16)*
4. **Of the men in question 13, how many did you know were HIV positive?**   *(If 0 please skip to question 16)*
5. **Of the men in question 14, how many did you know were on HIV treatment?**
6. **With how many different men have you been top (active) during anal sex in the last 90 days?**  *(If 0 please skip to question 20)*
7. **Of the men in question 16, with how many were you top without using a condom?**  *(If 0 please skip to question 20)*
8. **Of the men in question 17, how many did you know were HIV positive?**  *(If 0 please skip to question 20)*
9. **Of the men in question 18, how many did you know were on HIV treatment?**
10. **Of the men you’ve been either top or bottom with during anal sex in the last 90 days, how many were new partners?**  (This means men you had not had sex with before)
11. **Think of the last time you had anal sex (top or bottom) with a man without a condom. These are reasons other men have given for not using condoms, please tick all that apply.**

I don’t like using condoms

He doesn’t like using condoms

Condoms weren’t discussed

We don’t use condoms with each other but do with other partners

Neither of us had any condoms

I didn’t consider myself at risk of HIV

I was under the influence of alcohol

I was under the influence of drugs

I am faithful to him

He is faithful to me

It is more enjoyable without a condom

I was only dipping

Other _________

1. **Think of the last time you had anal sex (top or bottom) with a man without a condom. What was his HIV status?**

I don’t know

I thought he was HIV negative

I thought he was HIV positive and on treatment

I thought he was HIV positive and not on treatment

I thought he was HIV positive and did not consider whether he was on treatment

1. **In general, when you have anal sex (top or bottom) without using a condom to what extent do you consider yourself at risk of getting HIV?**

| Not applicable | No risk | A little risk |
| --- | --- | --- |
| Somewhat at risk | Large risk | Very large risk |

1. **In general, how do you manage your risk of getting HIV?** *(Tick all that apply)*  I frequently ask my partner to use a condom for anal sex

I frequently use condoms

I choose partners based on their negative HIV status

I seek partners who I know are on HIV treatment

I think about strategic positioning (I try to be top if I’m not sure about my partner’s HIV status)

I don’t think about these risk reduction strategies

1. **How many times in the past 12 months have you attended a clinic for a HIV test?**
2. **How many times in the past 12 months have you attended a clinic for a STI test?**
3. **How many times in the past 12 months have you been prescribed a course of post-exposure prophylaxis (PEP, taking antiretroviral (anti-HIV) drugs soon after potential HIV exposure for 4 weeks to reduce the risk of becoming infected with HIV)?**
4. **In the past 12 months have you been diagnosed with any of the following?**

|  | Yes | No |  | Yes | No |
| --- | --- | --- | --- | --- | --- |
| Rectal Gonorrhoea |  |  | Syphilis |  |  |
| Urethral Gonorrhoea |  |  | Hepatitis B |  |  |
| Oral Gonorrhoea |  |  | Hepatitis C |  |  |
| Rectal Chlamydia |  |  | Genital warts (new or recurrent) |  |  |
| Urethral Chlamydia |  |  | Genital herpes (new or recurrent) |  |  |
| Oral Chlamydia |  |  | Trichomonas |  |  |
| LGV |  |  |  |  |  |

**Health and wellbeing Questions**

| 1. **Over the last 2 weeks, how often have you been bothered by any of the following problems?** | | | | |
| --- | --- | --- | --- | --- |
|  | Not at all | Several days | More than half the days | Nearly every day |
| a) Little interest or pleasure in doing things |  |  |  |  |
| b) Feeling down, depressed or hopeless |  |  |  |  |
| c) Trouble falling or staying asleep, or sleeping too much |  |  |  |  |
| d) Feeling tired or having little energy |  |  |  |  |
| e) Poor appetite or overeating |  |  |  |  |
| f) Feeling bad about yourself – or that you are a failure or have let yourself or your family down |  |  |  |  |
| g) Trouble concentrating on things, such as reading the newspaper or watching television |  |  |  |  |
| h) Moving or speaking so slowly that other people could have noticed. Or the opposite being so fidgety or restless that you have been moving around a lot more than usual |  |  |  |  |
| i) Thoughts that you would be better off dead, or of hurting yourself in some way |  |  |  |  |

**Lifestyle Questions**

1. **In the past 3 months have you used recreational drugs (e.g. poppers, cannabis, cocaine)?**Yes  No  *(If no, go to question 32)*
2. **If yes, which drugs have you used?**

| Acid/LSD/magic mushrooms |  | Heroin |  |
| --- | --- | --- | --- |
| Anabolic steroids |  | Ketamine (K) |  |
| Cannabis (marijuana, grass) |  | Khat (chat) |  |
| Cocaine (coke) |  | Mephedrone |  |
| Crack |  | Morphine |  |
| Codeine |  | Opium |  |
| Crystal meth (methamphetamine) |  | Poppers (amyl nitrate) |  |
| Ecstasy (E) |  | Speed (amphetamine) |  |
| GHB (liquid ecstasy) |  | Viagra |  |
|  |  | Other (please specify)___________ | |

1. **How often have you had a drink containing alcohol (beer/wine/spirits/mixed drink) in the last 90 days?**

| Daily | Nearly every day | 3 or 4 times a week |
| --- | --- | --- |
| Once or twice a week | 2 or 3 times a month | Once |
| Never  *go to question 34* |  |  |

1. **How many units of alcohol do you drink on a typical day when you are drinking?**

*(One unit=half a pint of beer/cider or a small glass of wine or a single measure of spirits)*

1. **Other men have suggested the following reasons as motivation for taking part in this trial.** *Please tick all the statements that you agree with.*

I want to contribute to scientific research

I want to receive the regular sexual counselling to help me understand and reduce my risk

I feel pressured to have sex without a condom

My partner is already in this trial

Taking PrEP would reduce my risk of getting HIV

I will be able to have more sex without condoms

I want to help the gay community

Other _________

1. **PrEP will be prescribed to men participating in this trial, either now or in the future, as a DAILY medication. When you are offered PrEP how often do you think you’ll miss a tablet?**  I will find it easy to remember to take my drug daily

I might forget to take my pill at my scheduled time but will remember to take it within a few hours

I might occasionally forget to take a dose

I might forget to take my drug once or twice a week

I will remember to take my pill if I know I am going to be having sex in a couple of days

I will find a daily dosing schedule very difficult to follow

***Thank you for finishing the questionnaire.***

***Please place in the provided envelope and hand to a doctor or nurse***
