# Supplementary material for: An analysis of baseline data from the PROUD study: an open-label randomised trial of pre-exposure prophylaxis
Source: Trials. 2016 Mar 24;17:163. doi: 10.1186/s13063-016-1286-4 (PMC4806447; doi:10.1186/s13063-016-1286-4)
Supplement: Additional file 2: — Adherence and Sexual Behaviour Questionnaire. (DOC 95 kb) [file 13063_2016_1286_MOESM2_ESM.doc]

**PROUD**

| **Adherence and Sexual Behaviour Questionnaire** |
| --- |

| Trial no: | Initials: | Date of birth: | Date form completed: |
| --- | --- | --- | --- |

*This form should be completed monthly and just before your 3-monthly clinic visit; you will be sent a reminder.*

*Please bring all completed forms with you to you clinic appointment for discussion with your nurse/doctor.*

**Sexual Behaviour**

This section is about your sexual behaviour in general over the last 30 days. Unless otherwise specified “sex” means anal intercourse (including “dipping”), either as bottom (receptive, passive, he fucked you) or as top (insertive, active, you fucked him). Approximate answers are fine.

1. **How many different men did you have sex with in the last 30 days?**  *if ‘0’ go to q14 if currently prescribed PrEP tablets or to end of this questionnaire if you are not currently prescribed PrEP*
2. **With how many different men have you been a top during anal sex without a condom in the last 30 days?**
3. **With how many different men have you been a bottom during anal sex without a condom in the last 30 days?**  *if 0 go to question 7*
4. **Of the men in question 3, how many men did you not know their HIV status?**
5. **Of the men in question 3, how many did you think to be HIV positive and not currently on treatment?**
6. **Of the men in question 3, how many have you previously had sex with?**

**Recent Sexual history**

This section is about your recent sexual history. It can include partners who you have already included in question 3.

1. **Think of the last time you had anal sex in the last 30 days.***For each statement please tick one box on each row*
2. **I was top (active) during anal sex**No  Yes with a condom  Yes without a condom
3. **I was bottom (passive) during anal sex**No  Yes with a condom  Yes without a condom
4. **How many days ago was this?**  days
5. **Think of the last time you were bottom (passive) during anal sex without a condom.
   When was this?**  days ago
6. **What was the HIV status of this partner?**

I don’t know

I thought he was HIV negative

I thought he was HIV positive and on treatment

I thought he was HIV positive and not on treatment

I thought he was HIV positive and did not consider whether he was on treatment

1. **Was this a partner who you have previously had sex with?** Yes  No

*Please skip to the end of this questionnaire if you are not currently prescribed PrEP tablets or if you are currently prescribed PrEP tablets but have not had sex without a condom in the last 30 days go to qu14*

1. **In the 7 days before you had sex without a condom how many days did you miss PrEP tablets? 0** **1** **2**  **3**  **4**  **5**  **6**  **7**
2. **In the 7 days after you had sex without a condom how many days did you miss PrEP tablets? 0** **1** **2**  **3**  **4**  **5**  **6**  **7**  *If it has not yet been a full 7 days since you had sex without a condom please estimate how many PrEP tablets you expect to miss*

**Your experience of using PrEP**

Many people miss tablets for a variety of reasons

1. **Approximately what percentage of days in the last 30 have you missed PrEP tablets?** *Please mark on the line below*

10%

20%

30%

40%

50%

60%

70%

80%

90%

100%

0%

| 0  3  6  12  15  18  21  24  27  30  9  Missed NONE  Missed HALF  Missed ALL  tablets |
| --- |

1. **These are reasons other people have given for missing PrEP tablets*.* For each reason, please show how often they apply to you by ticking the box that fits**

| **Reasons for missing PrEP tablets** | Always | Often | Sometimes | Rarely | Never |
| --- | --- | --- | --- | --- | --- |
| a) I forgot |  |  |  |  |  |
| b) I am not convinced I needed PrEP |  |  |  |  |  |
| c) I decided to stop altogether |  |  |  |  |  |
| d) I am using other protection |  |  |  |  |  |
| e) I had a bad side effect to PrEP |  |  |  |  |  |
| f) I lost my PrEP tablets |  |  |  |  |  |
| g) I decided to give myself a break from PrEP |  |  |  |  |  |
| h) I did not have my PrEP tablets with me |  |  |  |  |  |
| i) I am not currently having sex |  |  |  |  |  |
| j) I was concerned that PrEP might be harmful |  |  |  |  |  |
| k) I don’t like the idea of taking PrEP all the time |  |  |  |  |  |

***Thank you for finishing the questionnaire.***

***Please place in the provided envelope and hand to a doctor or nurse during your next clinic visit.***
